# OdoBD: An online database for the dragonflies and damselflies of Bangladesh

**DOI:** 10.1371/journal.pone.0231727

**Published:** 2020-04-23

**Authors:** Md Nur Ahad Shah, Md Kawsar Khan

**Affiliations:** Department of Biochemistry and Molecular Biology, Shahjalal University of Science and Technology, Sylhet, Bangladesh; Instituto Federal de Educacao Ciencia e Tecnologia Goiano - Campus Urutai, BRAZIL

## Abstract

Combining scientific data over a long-time period is necessary for generating large-scale datasets, which are an essential component of comparative analysis for understanding evolutionary processes. Furthermore, monitoring temporal and spatial distributions of animals at a global and regional scale is essential for studying climate change driven extinction risks. Regional and global datasets focusing on different animal groups are on the rise to meet such challenges. Although being one of the earliest and best-known insect groups, the data on Odonata remains rudimentary and dispersed, especially in the South Asian region. Bangladesh, being located within a biodiversity hotspot, possesses a large number of odonate species and many of them are endemic to the South Asian region. We have developed an online database for the Odonata of Bangladesh by compiling and digitizing data from our last four years of field studies, from previously published research articles and field guides, and also by collecting data from citizen scientists. The Odonata of Bangladesh database (accessible at http://www.odobd.org) contains phenotypic, genotypic, photographic, taxonomic, biogeographic and faunistic data of the Odonata of Bangladesh. The database will be a valuable resource for understanding diversity, distributions, extinction risks and conservation planning of the Odonata of Bangladesh. Finally, phenotypic, spatial and temporal data of Odonata of Bangladesh datasets can be integrated with other regional datasets for analyzing macroevolutionary trends and to monitor the effect of climate change on odonates.

## Introduction

Scientific descriptions of natural organisms can be regarded as some of the most valuable documents in the study of historical occurrences of organisms. Museum records serve as a credible source of information about the diversity of biological organisms, and include voucher specimens, however, comparing and contrasting museum records from different geographical locations for large-scale analysis is an exceedingly difficult task. A variety of digital catalogs have been developed to confront this challenge. Consolidated online databases such as the Atlas of Living Australia (https://www.ala.org.au/), iNaturalist (http://www.inaturalist.org/), the IUCN Red List (http://www.iucnredlist.org/), and VertNet [1], with their geographical, physiological and biochemical information, are now essential sources for large-scale analysis [2]. The online databases focusing on invertebrates, especially insects, are lagging far behind [3]. However, in response to growing needs, online invertebrate databases are gradually increasing in number as more insect data are being digitized in online repositories such as FreshWaterBiodiversity (http://data.freshwaterbiodiversity.eu/), Global Biodiversity Information Facility (http://www.gbif.org/), OdonataCentral [4], and the Odonate Phenotypic Database [5]. Along with these online databases containing worldwide information, regional databases like Butterflies of India (https://www.ifoundbutterflies.org/), Butterflies of Belgium [6], and Odonata of India (https://www.indianodonata.org) are currently emerging, providing more detailed insights on the extant species with their spatial and temporal information.

The order Odonata is one of the earliest and best-known insect groups, existing on all continents except Antarctica [7]. These insects predominantly inhabit the tropical and subtropical climate zones [8]. Adult Odonates are terrestrial in nature, found adjacent to water sources, whereas the immature stages are aquatic, inhabiting freshwater habitats of all kinds, ranging from permanent running waters like rivers and lakes to small temporary rain pools and puddles. Being a species specific to a certain type of habitat makes them an ideal candidate for monitoring the health of freshwater ecosystems. The taxonomic Order Odonata is divided into three suborders–Anisoptera, which encompasses dragonflies; Zygoptera, which includes damselflies; and Anisozygoptera, which contains intermediary species between these two groups. Currently, about 6400 different species of Odonates within 600 genera have been described globally [5,9]. A combined effort has been undertaken to enlist the Odonata of the world, in order to make them readily available to the interested scientific community [9]. Additionally, there have been region-specific studies on the odonates in different parts of the world, specifically countries with a diverse range of Odonata [10,11].

Bangladesh is a small country with high Odonata diversity. Currently, more than a hundred species are known from Bangladesh. The largely unconsolidated information makes large-scale analysis and research involving Bangladeshi Odonates particularly challenging. Thus, we have developed an online database of all the known Odonates from Bangladesh to provide an integrated and widely accessible source to facilitate studies of evolution, ecology, taxonomy, and conservation. Currently, we have amassed information about 102 different species from all over the country. The Odonata of Bangladesh database (accessible at http://www.odobd.org), contains data on morphology, abundance, flight season, gene and protein sequences, local and global distribution and conservation status, and gender specified photographs. Bangladeshi Odonates and is updated on a regular basis. This database is a rich resource of diversity and distribution data for Bangladeshi Odonata and will be an essential component to understand their extinction risks and to develop conservation demands. Furthermore, this database, along with other datasets, could be used in global analysis for understanding macroecological patterns and the impact of climate change.

## Methods

### Ethical statement

We collected data mainly from previously published articles for which we did not require ethics approval. Furthermore, we did not conduct fieldwork in any protected areas or national parks and none of the studied species were endangered or protected, therefore we did not require animal ethics permission for this study.

### Data resources

**Fig 1:**
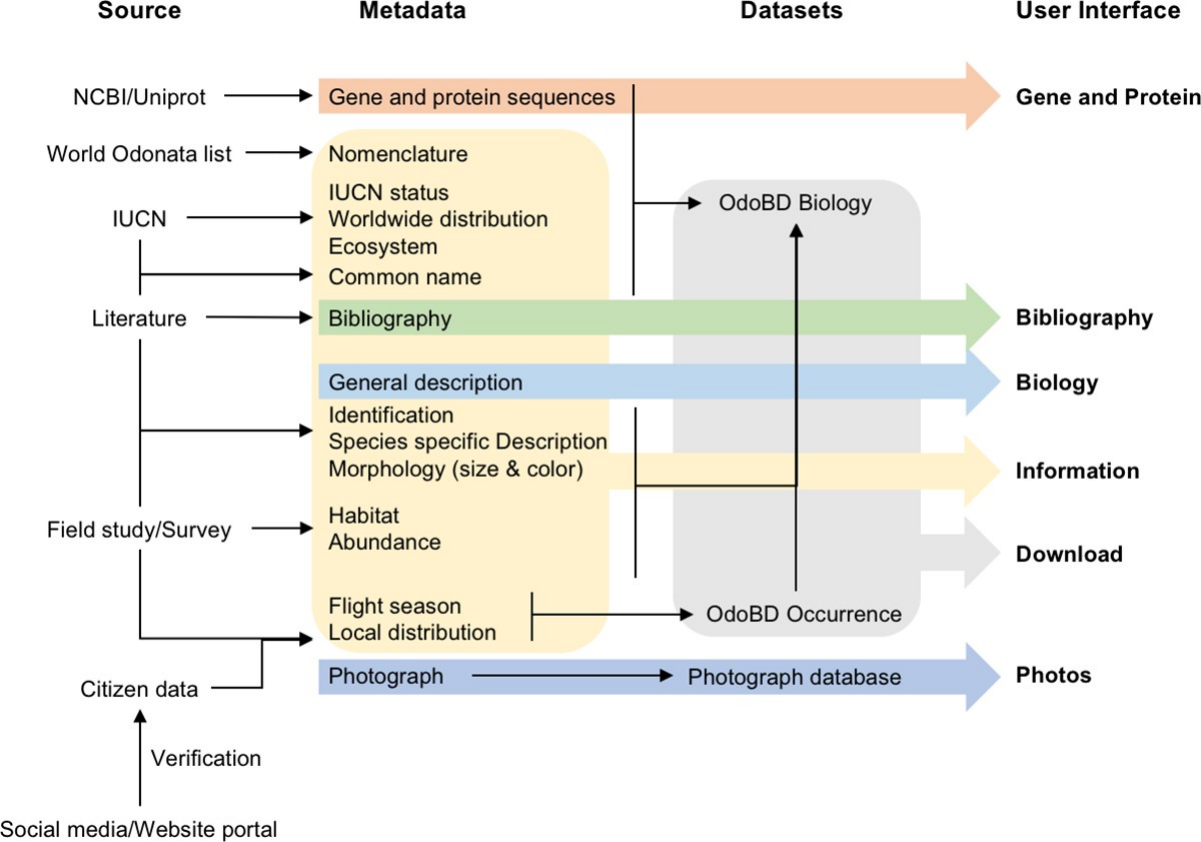
Schematic diagram of the database showing the sources and the structure of the data in relation to the user interface.

The list of the Odonata species of Bangladesh was compiled from previously published articles [12–15]. We followed the World Odonata list (https://www.pugetsound.edu/academics/academic-resources/slater-museum/biodiversity-resources/dragonflies/world-odonata-list2/) for nomenclature of the species, and Dijkstra et al., 2013 for taxonomical classifications [16]. Common name, preferred ecosystem, global conservation status, and global distribution data were collected from the International Union for Conservation of Nature and Natural Resources database (http://www.iucnredlist.org/). Phenotypic data were aggregated from previously published articles [17–21] and field guides of the South Asian region [22]. Geographical distribution, occurrence records, abundance, and flight season data were extracted from previously published articles and also from our unpublished data from fieldwork [12–15]. As genomic and proteomic studies of odonates are limited, the number of sequenced genes and proteins is very low. We included the most sequenced gene in the odonates - cytochrome oxidase and its corresponding protein cytochrome c oxidase (EC 1.9.3.1) - for genomic and proteomic data users. The gene and protein sequences for the species were collected from the National Center for Biotechnology Information database (http://www.ncbi.nlm.nih.gov/) and the UniProt database (http://www.uniprot.org/). Data resources are graphically presented in Fig 1, and a list of resources used in this study is provided in S1 File.

### Geographic coverage and occurrence data

**Fig 2:**
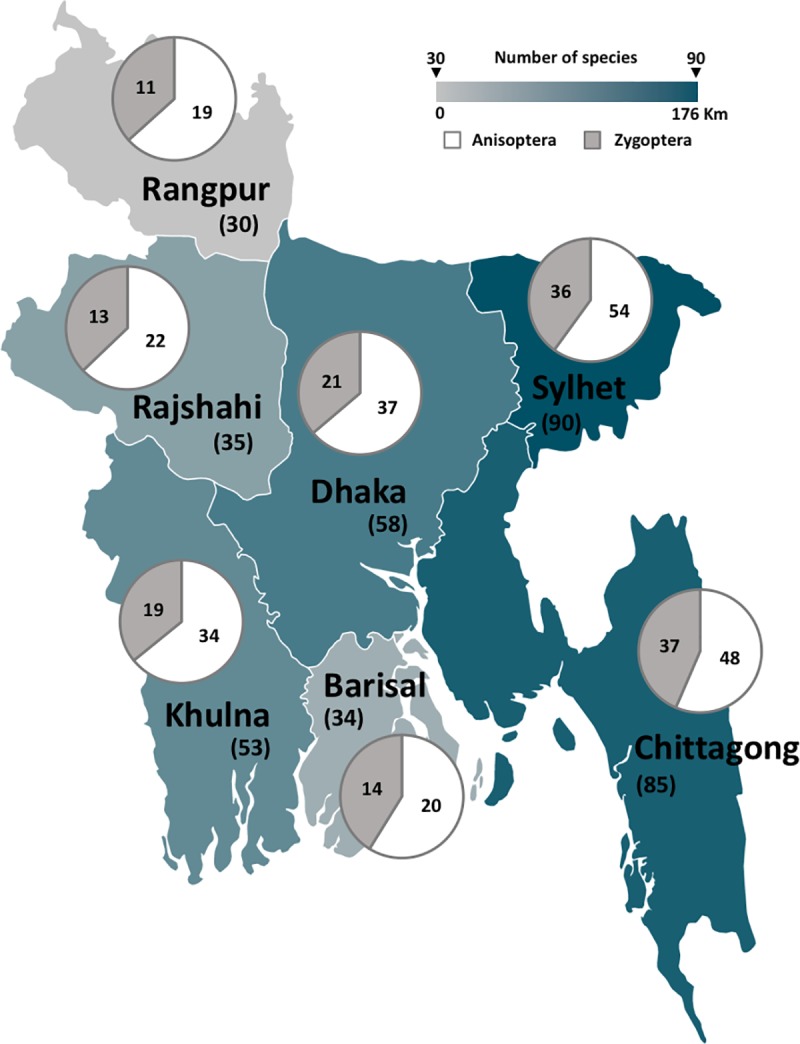
A reference map of different divisions of Bangladesh with the number of extant Odonata species in that region. Sub-order Anisoptera is represented in white and Zygoptera is represented in grey.

The database Odonata of Bangladesh compiled distribution data of the Bangladeshi odonates from the whole country. For the development of the database, we have divided the country into seven major regions, namely Barisal, Chittagong, Dhaka, Khulna, Rajshahi, Rangpur, and Sylhet (Fig 2). Our study was mainly focused on four specific regions serving as Odonata breeding hotspots–Dhaka, Sylhet, Chittagong, and Khulna; which correspond to the central, north-eastern, south-eastern and south-western part of the country, respectively. These regions encompass nearly all of the distinct climates and water bodies of Bangladesh. We did regular surveys throughout the year for four years (2012–2016) in those regions and also conducted occasional surveys on the rest of the country, parts of which have been published previously [12–15].

We aggregated distribution data of the Bangladeshi Odonata from published articles, from our unpublished studies, and from the data deposited by citizen scientists in our social media platform (www.facebook.com/groups/OdoBD) and website portal. We extracted species-specific geographical coordinates when they were provided in the publications. Otherwise, we determined the longitude and latitude of a location using google maps (www.google.com/maps) [23]. When administrative districts or provinces were reported as occurrence locations, we extracted longitude and latitude of the center of the region [23]. We further classified all occurrence data into three levels according to their administrative regions (i.e. division, district, and finer level). This classification will assist users to extract sections according to their interests.

### Phenotype and photographs

We extracted phenotypic data of Odonata of Bangladesh from previously published field guides [17–20]. We extracted body size data such as abdominal length and wing length of males and females. We also used other phenotypic characters that might be of interest for taxonomic, ecological and evolutionary studies such as identification features, wing spots, and body color.

We captured most of the photos deposited on the database in the last four years (2012–2016) using a Canon 600D camera with a 55-250 mm lens. These photographs focus on various identification features of the dragonflies. For species that are difficult to identify, we collected specimens by using an insect sweeping net for proper identification. Most of the photos were taken in natural habitats between 0800 and 1700 hours. We also collected photographs from citizen scientists using our website portal, and deposited them in the database, crediting the photographers. We captured photographs of males and females, their color morphs, and body-color at different developmental stages. We also took photographs of their life history such as emerging, perching, foraging, mating, oviposition, territorial fight etc.

### Flight season

Seasonality is an important factor for studying invertebrates, especially for insects with short life cycles. We, therefore, extracted the time of the year when a species was recorded from previously published articles and also from our unpublished articles. Due to its location, Bangladesh has a temperate climate with six seasons, each of which is comprised of two months. The six seasons and their ranges are: Summer (April-May), Monsoon (June-July), Autumn (August-September), Late Autumn (October-November), Winter (December-January) and Spring (February-March). Based on the amassed occurrence records, we have presented the flight months and season of every odonate found in Bangladesh.

### Data accuracy and accessibility

The Odonata of Bangladesh database consists of two datasets: 1) OdoBD_occurrence contains occurrence data and 2) OdoBD_biology contains all other data. Both authors entered data on these datasets. Both authors then double-checked all entries and corrected any errors. These two datasets can be downloaded directly as csv files from the website (www.odobd.org/download/), or from the Dryad data repository [24]. We will continue to update and correct the database based on feedback from peer reviewers and users.

Further information on the online database structure and usage notes are presented in S2 File.

## Results

### Taxonomic coverage

The Odonata of Bangladesh contains data for a total of 102 species, of which 57 were Anisoptera (dragonflies) from four families, and 45 were Zygoptera (damselflies) from six families. Libellulidae and Coenagrionidae were the highest represented Anisopteran and Zygopteran families in our database with 45 and 27 species respectively. Currently, photographs of 84 males and 54 females are deposited in the database.

### Distribution and conservation status

Among the seven regions, Sylhet and Chittagong were found to have the most species diversity. A total of 90 species (54 dragonflies and 36 damselflies) occurred in Sylhet, whereas 85 species (48 dragonflies and 37 damselflies) occurred in Chittagong (Fig 2). Dhaka and Khulna had a moderate level of Odonata diversity, with total sightings of 58 and 53 species respectively (Fig 2). The remaining three regions had a lower number of species sightings: Rajshahi 35, Barisal 34, and Rangpur 30 (Fig 2).

The IUCN Red List status analysis showed that, of the documented 100 species, 92 belong to the Least Concern (LC) category. 51 of these species were Anisoptera and 41 were Zygoptera. Seven species were recorded under the category of Data Deficient (DD), five of which belong to the sub-order Anisoptera (Aeshnidae 2 and Gomphidae 3), and two species (*Dysphaea walli* and *Mortonagrion varralli*) belong to the sub-order Zygoptera. One documented species, *Indothemis carnatica*, is designated as Near Threatened (NT). The rest of the two Zygoptera species, namely *Matrona nigripectus* and *Agriocnemis kalinga*, have not yet been assessed by IUCN.

### Flight season

**Fig 3:**
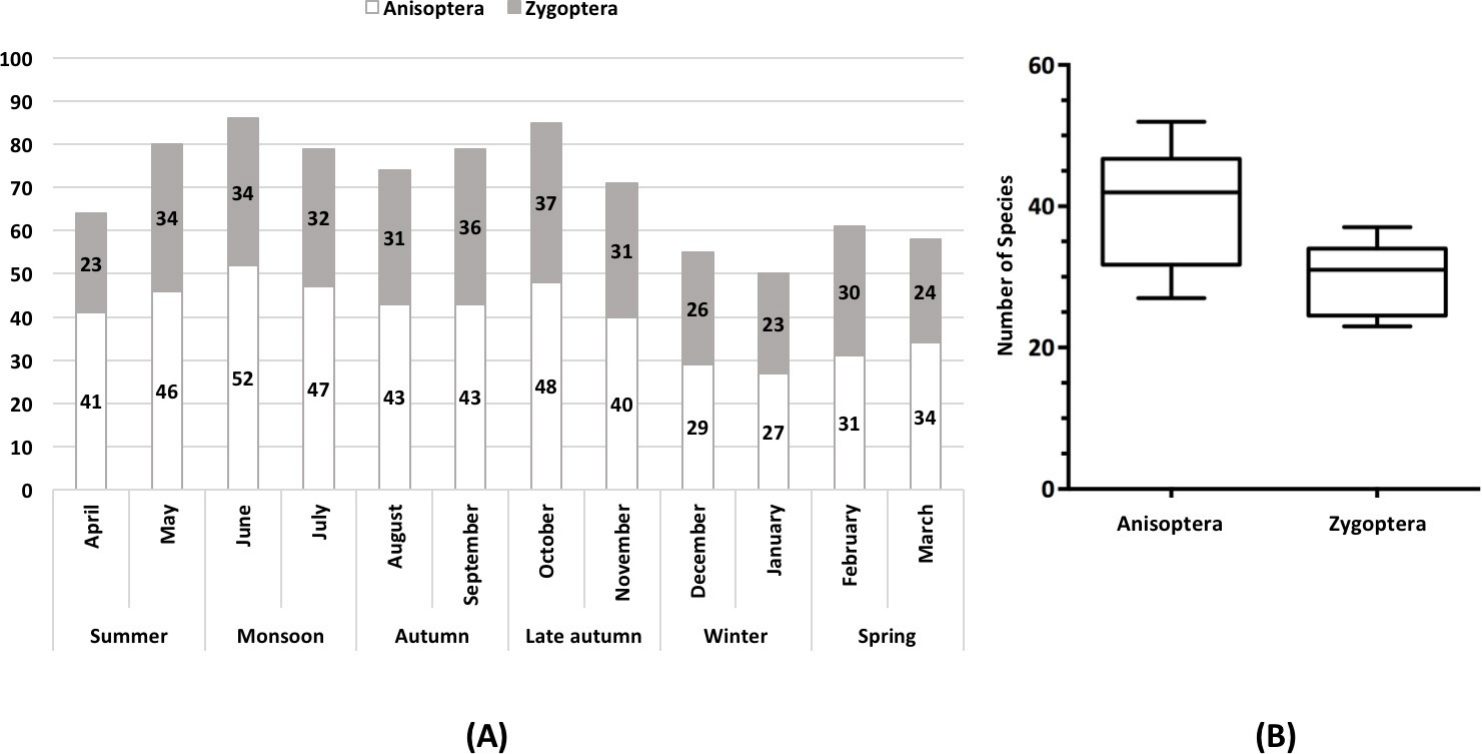
A) Number of Odonata species based on their flight pattern in different seasons of Bangladesh. White bars represent the sub-order Anisoptera and grey bars represent the sub-order Zygoptera. B) A boxplot diagram of the encountered Odonata species throughout the year.

A total of 34 species were found to be abundant all year long. Odonata sightings start to peak in the month of May, which is the mid-summer season, and continue until November, which is the end of late autumn (Fig 3A). Their prevalence then starts to decline, with fewest sightings during the winter season. The number of Anisopteran species observed per month ranges from 27 (January) to 52 (June), and Zygopteran observations range from 23 (January/April) to 37 (October) (Fig 3B).

### Data records

#### OdoBD biology dataset

All data on the odonates of Bangladesh were compiled into two datasets. The OdoBD biology dataset contains all metadata. Each row of the dataset represents data for a single species, and the columns contain different variables (Table 1).

**Table 1:** Data structure and variable definition of OdoBD_biology dataset.

Column label	Column description
**id**	Unique identification number
**suborder**	Suborder of the species
**family**	Family of the species
**genus**	Genus of the species
**specificname**	Specific name of the species
**scientist**	Name of the first identifying scientist
**year**	Year of the first identification
**commonname**	Common name of the species
**IUCN_status**	IUCN status of the species
**habitat**	Preferred habitat of the species
**system**	Eco-system of the species
**distribution**	Local distribution of the species
**abundance**	Relative abundance of the species
**worldwide_distribution**	Worldwide distribution of the species
**flightseason**	Preferred flight season
**description**	General description
**desmale**	Male description
**desfemale**	Female description
**identification**	Identification features of the species
**COI_gene**	Genetic sequence of cytochrome oxidase gene
**co1_protein**	Protein sequence of cytochrome c oxidase
**male_abdomen**	Length of male abdomen size in mm
**female_abdomen**	Length of female abdomen size in mm
**male_wing_size**	Length of male wing size in mm
**female_wing_size**	Length of female wing size in mm
**wing_spot**	Male and female wing spot
**eye**	Male and female eye color
**key**	Identification features to distinguish species within same family

#### OdoBD occurrence dataset

The OdoBD occurrence dataset contains occurrence and temporal data of the Odonata of Bangladesh. At present, the dataset contains 4525 occurrence records of 102 species from 240 different locations and 59 administrative districts from January 1994 to December 2019. Among them, we collected 950 occurrence data for 74 different species from 140 different locations from citizen scientists from August 2009 to December 2019. In all subsequent analyses, species that are highly unlikely to occur in a particular region or reported as a possible misidentification were considered as vagrant and were kept in a separate table (S3 File).

Each row of the dataset represents a single record (an occurrence of an Odonata species recorded in a specific year from a location with a source reference) and columns encompass different variables (Table 2).

**Table 2:** Data structure and variable definition of OdoBD_occurrence dataset.

Column label	Column description
**id**	Unique identification number
**suborder**	Suborder of the identified species
**family**	Family of the identified species
**species**	Specific name of the identified species
**division**	Division level information of the location
**district**	District level information of the location
**coordinates**	The longitudinal and latitudinal coordinates of the location of occurrence
**date**	Month and year of the observation (mm-yy)
**source**	Reference for the data source

## Discussion

The storage and access of large datasets for scientific research can be achieved by means of universal electronic databases. Such databases have revolutionized large scale ecological and evolutionary analyses and are essential components for understanding macroevolutionary processes. Global, regional and national databases are continuously being developed to meet such demands. However, no such database is available for the Odonata of Bangladesh. We have developed a comprehensive database for the dragonflies and damselflies of Bangladesh. We have aggregated phenotypic (body size, wing size, body and wing color), taxonomic (photographs, description, gene and protein sequences, and identification keys), biogeographic (regional and global distribution), and faunistic data of the Odonata of Bangladesh.

Comparative analysis is an essential part of understanding phenotypic trait evolution, which requires large phenotypic datasets [25,26]. Odonata body and wing size have been studied previously to understand the relationship between size and geographic latitude [27,28]. Furthermore, a recent study with a small number of Odonata has shown that body size is correlated with extinction risk [29]. Our database provides body and wing size data of 103 species which would facilitate future studies on micro- and macro-evolutionary patterns of body size evolution and extinction risk determination. Moreover, our database possesses male and female specific size data which could be suitable for determining how sexual size dimorphism is correlated with latitude and mating systems, as these characteristics have previously been shown to influence sexual size dimorphism [30].

With the advent of modern Geographical Information Systems (GIS), it is now possible to amass large-scale animal occurrence data that can help us to understand the habitat requirements of species. Temporal data combined with occurrence data can further aid us in understanding the impact of climate change [31,32]. This is particularly important for the Odonata–a family of species that has a short life cycle and requires a strict habitat. We, for the first time, provided an occurrence and temporal dataset for the Odonata of Bangladesh. These data will accommodate studies, such as species distribution modeling, which improve our understanding of the habitat requirements of Odonata species and hence assess their extinction risks. We will continually update the database to observe how temporal changes influence the community structure, habitat integrity, and extinction risks of the odonates.

Digital photographs are a powerful medium to study the functional, ecological and evolutionary significance of animal coloration [33]. Recent studies have exhibited the importance of photographic websites for studying the ecology and evolution of animal coloration [34,35]. We deposited photographs of males and females of the Bangladeshi Odonata in our database. These photos will provide a powerful tool to study sexual dichromatism, which is a sexually selected trait in odonates [36,37]. Furthermore, many odonates exhibit color polymorphism, which reduces sexual conflict and increases population fitness [38,39]. Our deposited photographs of different color morphs could be applied to color polymorphic studies. Finally, the photographs of odonates at different developmental stages would assist in studies of ontogenetic color, which has been linked to the signaling of sexual maturity and reduction in sexual harassment [40,41].

We have developed a database of the Odonata of Bangladesh by digitizing data from previously published articles, field guides, and from our unpublished works. We have amassed phenotypic, taxonomic, biogeographic, and faunistic data of the dragonflies and damselflies of Bangladesh. The database will be a valuable resource for understanding the ecology, extinction risks and conservation importance of the Bangladeshi odonates. Furthermore, this database could be useful for comparative analysis to understand the macroecological and biogeographical patterns and processes. Our deposited photographs of males and females, their developmental stages, and the color morphs of different species will be a valuable resource for studying the function of color in odonates. We will continue updating our database by adding temporal and occurrence data which will help to monitor the impact of climate change and extinction risks of the Odonata of Bangladesh.

## Supporting information

S1 File (CSV)

S2 File (DOCX)

S3 File (CSV)
