# Supplementary material for: OdoBD: An online database for the dragonflies and damselflies of Bangladesh
Source: PLoS One. 2020 Apr 23;15(4):e0231727. doi: 10.1371/journal.pone.0231727 (PMC7179912; doi:10.1371/journal.pone.0231727)
Supplement: S2 File — (DOCX) [file pone.0231727.s002.docx]

**Database structure and usage notes:**

The website Odonata of Bangladesh database is accessible at [www.odobd.org](http://www.odobd.org). The MySQL database runs on a Percona server (webserver cpsrvd ver.11.x). Data are stored in the relational tables of a MySQL ver. 5.x database. A graphical user interface (GUI) named phpMyAdmin was installed in the server for managing MySQL tables and data. The GUI is accessible through all class of browsers regardless of operating system, though it has been most intensively tested using Mozilla Firefox and Google Chrome.

The following data are deposited for each accession: Taxonomy, classification, general information (common name, scientific name, abundance, flight season, local and global distribution, and IUCN status, description, and gene and protein sequences), photographs (male, female, foraging and reproductive behavior such as copula, oviposition, tandem when available). A map of local distribution of each species is also included. General information on the difference between dragonfly and damselfly, their morphology, habitat, reproductive behavior, predator and prey interrelationship and conservation status are included under category ‘Biology’. The bibliography section contains a list of the previously published article on the Odonata fauna of Bangladesh. A common portal was created for citizen scientists to interact with the OdoBD database management team to submit sightings of the Odonata of Bangladesh. A static version of the database is accessible via Dryad (doi: 10.5061/dryad.j6q573n9c) and can also be downloaded from the website (www.odobd.org/download/).
